# Stress Hormones: Emerging Targets in Gynecological Cancers

**DOI:** 10.3389/fcell.2021.699487

**Published:** 2021-07-09

**Authors:** Guoqiang Chen, Lei Qiu, Jinghai Gao, Jing Wang, Jianhong Dang, Lingling Li, Zhijun Jin, Xiaojun Liu

**Affiliations:** ^1^Department of Obstetrics and Gynecology, Changzheng Hospital, Naval Medical University, Shanghai, China; ^2^School of Pharmacy, Naval Medical University, Shanghai, China

**Keywords:** perineural invasion, noradrenaline, epinephrine, dopamine, neurotrophic factors, glucocorticoids, tumor immune microenvironment

## Abstract

In the past decade, several discoveries have documented the existence of innervation in ovarian cancer and cervical cancer. Notably, various neurotransmitters released by the activation of the sympathetic nervous system can promote the proliferation and metastasis of tumor cells and regulate immune cells in the tumor microenvironment. Therefore, a better understanding of the mechanisms involving neurotransmitters in the occurrence and development of gynecological cancers will be beneficial for exploring the feasibility of using inexpensive β-blockers and dopamine agonists in the clinical treatment of gynecological cancers. Additionally, this article provides some new insights into targeting tumor innervation and neurotransmitters in the tumor microenvironment.

## Introduction

Cervical cancer and ovarian cancer are two major gynecological malignancies. Preliminary and secondary strategies for the prevention of cervical cancer have reduced its rates of incidence and mortality. However, in 2018, there were 106,000 cases of cervical cancer in China and 48,000 deaths (Arbyn et al., 2020). Therefore, cervical cancer remains the second leading cause of cancer-related death among young and middle-aged women (Bray et al., 2018; Siegel et al., 2020). Ovarian cancer is the seventh most common cause of cancer and the eighth leading cause of death in women. As ovarian cancer is difficult to diagnose early and is associated with high malignancy and drug resistance, it has the worst prognosis and highest mortality rate among all gynecological cancers (Coburn et al., 2017; Webb and Jordan, 2017; Torre et al., 2018). Therefore, a better understanding of the biological behaviors of cervical cancer and ovarian cancer is urgently needed, and novel therapeutic targets need to be identified.

Perineural invasion (PNI) has emerged as a novel research hotspot and is a harbinger of a poor prognosis in multiple cancers, including cervical cancer and ovarian cancer. Cervical cancer and ovarian cancer promote their own PNI via the release of neurotrophins (Allen et al., 2018; Long et al., 2018), axonal guidance molecules (Madeo et al., 2018), and exosomes (Madeo et al., 2018; Lucido et al., 2019; Vermeer, 2019; Kovacs et al., 2020). In addition, Schwann cells and cervical cancer cells can work in concert to promote tumor innervation (Huang et al., 2020). Evaluations of clinical specimens have also confirmed the presence of innervation in cervical cancer and ovarian cancer (Lucido et al., 2019; Kovacs et al., 2020; Reavis et al., 2020). In these evaluations, PNI in cervical cancer has a detection rate of 7.0% to 35.1% (Zhu et al., 2018; Zhu et al., 2019). Furthermore, existing studies suggest that there is a positive correlation between chronic stress and cancer progression. Long-term stress stimulation activates the sympathetic nervous system (SNS) and the hypothalamic-pituitary-adrenal axis (HPA), leading to the release of stress hormones, especially catecholamines and glucocorticoids. Catecholamine hormones can be further divided into norepinephrine (NE), epinephrine (E), and dopamine. These hormones act on β-adrenergic receptors, dopamine receptors (DRs), and glucocorticoid receptors. The interactions between stress hormones and receptors can produce a series of physiological effects on tumor cells and stromal cells.

The β-adrenergic receptors (β1, β2, β3) are a group of G protein-coupled receptors that mediate SNS signal transduction and activate downstream signaling pathways to prepare the body for “fight or flight.” β2-Adrenergic receptor (ADRB2) is overexpressed in ovarian cancer and cervical cancer and is positively correlated with a poor prognosis in patients (Lutgendorf et al., 2009; Huang et al., 2016; Chen et al., 2017). Ovarian cancer patients with high glucocorticoid receptor expression also have shorter progression-free survival and overall survival (Veneris et al., 2017; Veneris et al., 2019). The DRs include DR1 and DR2, both of which are highly expressed in ovarian cancer (Peters et al., 2020). Currently, no evidence has directly demonstrated that intratumoural infiltrating nerves are involved in the effect of stress on tumor cells. However, we hypothesize that under chronic stress, tumor innervation and receptors on the tumor cell surface may function via stress hormones to establish cross-talk and promote tumor progression together.

## Epidemiological Studies

Epidemiological studies have reported that depression, social isolation, and posttraumatic stress disorder, which cause long-term activation of the SNS, are closely related to the incidence of ovarian cancer. In patients with high depressive symptoms and low social support, the levels of NE in ovarian cancer tissues are significantly increased, and the risk of ovarian cancer or cancer progression is increased (Lutgendorf et al., 2009, 2011; Huang et al., 2015; Roberts et al., 2019). In contrast, eudaimonic well-being is negatively correlated with the NE levels in ovarian cancer tissues. Improving the eudaimonic well-being of patients with ovarian cancer has certain physiological protective effects (Davis et al., 2015). Although the specific mechanism has yet to be clearly elucidated, the possible explanation is that in ovarian cancer, the levels of circulating NE or intratumoural NE gradually increase due to the presence of chronic stress, which causes tumor vascularization, metastasis, invasion, and other effects.

Continuous human papillomavirus (HPV) infection is the main reason for the occurrence and development of cervical cancer. Severe types of stress, such as bereavement (loss of a parent, spouse, or child), may increase the risk of cancers related to HPV infection, such as cervical cancer. Continuous exposure to these severely stressful life events can increase the susceptibility of the host to cancer-causing HPV infection or accelerate the occurrence of established infectious cancers and ultimately lead to cervical cancer (Coker et al., 2003; Fang et al., 2011; Lu et al., 2016, 2019). Although behavioral changes after stressful life events may also play a role in cervical cancer, chronic stress-induced neuroendocrine disorders leading to changes in the biological behavior of tumor cells have been increasingly considered to be one of the biological mechanisms linking psychological stress with the occurrence and development of cervical cancer (Kennedy et al., 2014). Hence, regardless of the cause of cervical cancer, psychotherapy may be an important part of its prevention or treatment.

## Noradrenaline and Epinephrine

In response to stress, the levels of circulating catecholamines will increase. However, the local sympathetic nerve appears to provide most of the catecholamine content in tumor tissue, as we did not find any significant difference in circulating NE levels among tumor patients, nor did we find a significant correlation between plasma NE levels and intratumoural NE levels. However, these studies also had some limitations. Blood sampling was performed 2∼3 h before surgery, so parallel analyses of NE levels in the tumor and plasma could not be performed (Lutgendorf et al., 2009, 2011; Cole et al., 2015). In another study, mice were treated with hexamethonium bromide, a compound that can block ganglionic transmission in the peripheral nervous system. As expected, hexamethonium bromide completely eliminated the effect of stress on tumor growth. Tumor samples from animals that routinely faced restraint stress had significantly more innervation than tumor samples from control animals, and this increase could also be completely blocked by hexamethonium bromide. Adrenalectomy also failed to significantly inhibit stress-induced tumor growth, intratumoural nerve counts, and blood NE levels (Allen et al., 2018). All these results confirm the role of nerve endings in catecholamine-mediated tumor growth. Therefore, we concluded that under chronic stress, nerves in the tumor parenchyma can release neurotransmitters, such as NE and E, into the tumor microenvironment. Then, these neurotransmitters bind to receptors on the tumor cell surface and produce a series of effects on tumor cells. The effects are described below.

### Activation of Oncogenes

**FIGURE 1:**
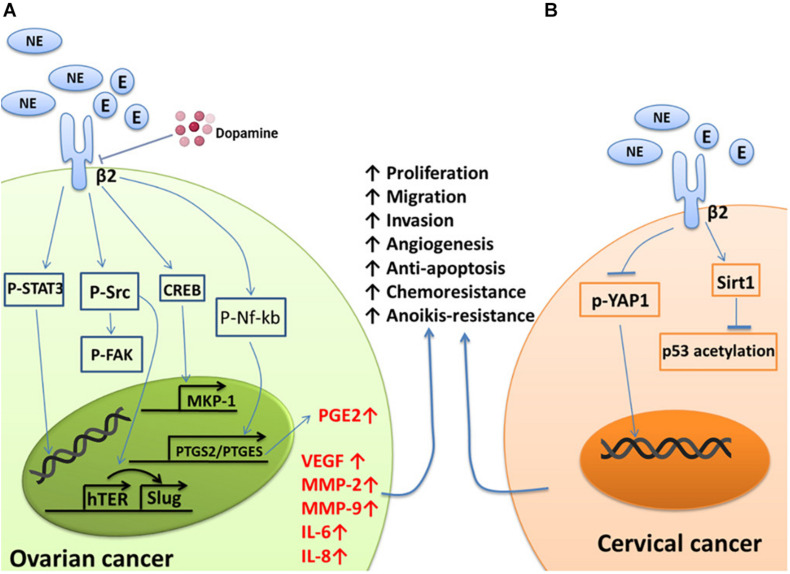
Summary of the effects of NE/E on pathways involved in cancer cell survival, metastasis, and chemoresistant signaling. **(A)** NE binds to ADRB2 to activate Src, which then induces the phosphorylation of FAK and the expression of VEGF, IL-6, and IL-8, conferring anoikis resistance, metastasis, and angiogenesis in ovarian cancer cells. STAT3 is phosphorylated and translocates to the nucleus to transactivate the target genes MMP-2 and MMP-9. NE can also activate ADRB2 to transcriptionally activate PTGS2 and PTGES via Nf-kb to produce PGE2. Finally, NE can induce ovarian cancer cells to become resistant to chemotherapy by acting on a target gene to induce MKP-1 expression through CREB. Additionally, NE-mediated tumor growth and angiogenesiscan be blocked by dopamine. **(B)** YAP1 is dephosphorylated and translocates from the cytoplasm to the nucleus in response to NE signaling, which results in anoikis resistance, a process initiated by the activation of ADRB2. Norepinephrine can also activate ADRB2 to induce chemoresistance by suppressing the acetylation of p53 through the upregulation of Sirt1 in cervical cancer cells.

The increases in the levels of NE and E induced by chronic stress can act on ADRB2 to promote tumor cell growth, metastasis, and angiogenesis (Sood et al., 2006; Thaker et al., 2006; Hassan et al., 2013; Cole et al., 2015; Jiang et al., 2020). These effects involve the activation of multiple tumor genes, including Src and signal transducer and activator of transcription-3 (STAT3). The Src protein plays important roles in the regulation of cell growth and differentiation, but abnormal activation of the Src protein is closely related to the occurrence of several tumors. Elevated NE levels lead to the abnormal phosphorylation of Src through ADRB2, followed by regulation of downstream pathways to enhance the proliferation, migration, and angiogenesis of ovarian cancer cells (Nilsson et al., 2007; Sood et al., 2010; Armaiz-Pena et al., 2013; Choi et al., 2015; Cole et al., 2015). It has also been confirmed that there is a positive correlation between high levels of NE in tumors and high Src phosphorylation levels in ovarian cancer tissues (Armaiz-Pena et al., 2013). STAT3 is another important oncogene. Abnormal activation of STAT3 triggers a variety of pathological events, including tumorigenesis (Calo et al., 2003). Norepinephrine and E induce STAT3 phosphorylation through ADRB2; STAT3 then translocates into the nucleus to activate target genes, leading to the proliferation, infiltration, and metastasis of ovarian cancer cells (Landen et al., 2007). Mitogen-activated protein kinase phosphatase-1 (MKP-1), also known as DUSP1, participates in the inactivation of MAPK and leads to the inhibition of apoptosis. High expression of MKP-1 is related to resistance to chemotherapy in ovarian cancer (Denkert et al., 2002). NE activates the cAMP-PKC-CREB signaling pathway through ADRB2 to induce the expression of the MKP-1 gene, which inhibits the responsiveness of ovarian cancer cells to paclitaxel chemotherapy (Wu et al., 2005; Kang et al., 2016). NE and E can also upregulate the expression of silent information regulator-1 (Sirt1) by activating ADRB2. Sirt1 can block the acetylation of p53, thereby conferring chemotherapy resistance to cervical cancer cells (Reed and Quelle, 2014; Chen et al., 2017) (Figure 1).

### Metastasis, Invasion, and Epithelial-Mesenchymal Transition

Metalloproteinase (MMP)-2 and MMP-9 play key roles in the invasion of malignant tumors (Davidson et al., 1999; Bergers et al., 2000; Huang et al., 2002; Sood et al., 2004). Norepinephrine and E can directly increase the invasive ability of ovarian cancer cells through upregulation of MMP-2 and MMP-9 via ADRB2. Propranolol (a non-selective β-blocker) can block this process (Sood et al., 2006; Thaker et al., 2006). In addition to being an inflammatory mediator, prostaglandin E2 (PGE2) is related to tumor cell proliferation, metastasis, and angiogenesis. Norepinephrine and E induce Nf-kb phosphorylation through ADRB2, and then p-Nf-kb enters the nucleus and binds with the PTGS2/PTGES gene to increase the synthesis of PGE2, which ultimately drives the proliferation and metastasis of ovarian cancer (Nagaraja et al., 2016). Epithelial-mesenchymal transition (EMT) plays an important role in embryonic development, damage repair, and cancer metastasis. Upregulation of the expression of Slug is an EMT hallmark (Hajra et al., 2002; Onder et al., 2008; Casas et al., 2011; Villarejo et al., 2014). Human telomerase reverse transcriptase (hTERT), apart from stabilizing the length of telomeres, is believed to promote malignant transformation independent of telomere lengthening. Norepinephrine upregulates hTERT-mediated Slug expression through Src and ultimately promotes the occurrence of EMT in ovarian cancer (Choi et al., 2015) (Figure 1A).

### Angiogenesis

Angiogenesis refers to the formation of new blood vessels by original endothelial cells and is an important physiological process in the repair of tissue damage. In cancer, angiogenesis is a key process for the growth and metastasis of most solid tumors, as it ensures a supply of oxygen and nutrients to the tumor tissue and transports metabolic waste from the tumor microenvironment (Lim et al., 2020). Although tumor angiogenesis is mainly driven by vascular endothelial growth factor (VEGF), it is also affected by MMPs, interleukin (IL)-6, IL-8, and so on. Norepinephrine can increase the expression of VEGF in ovarian cancer cells (Lutgendorf et al., 2003; Thaker et al., 2006; Chakroborty et al., 2009; Szubert et al., 2016) and promote the migration of endothelial cells by inducing the expression of MMP-2 and MMP-9 (Bergers et al., 2000; Huang et al., 2002; Thaker et al., 2006; Landen et al., 2007; Gonzalez-Villasana et al., 2015), thereby inducing the formation of new blood vessels in tumors. The cytokines IL-6 and IL-8 are vital in inflammation and can increase tumor angiogenesis (Browning et al., 2018; Taher et al., 2018; Kim, 2020; Fousek et al., 2021). Norepinephrine can induce ovarian cancer cells to produce IL-6 and IL-8 through effects on the Src protein and FosB protein, respectively, and thus promote angiogenesis in ovarian cancer (Nilsson et al., 2007; Shahzad et al., 2010) (Figure 1A).

### Cell Survival

Anoikis refers to the process of programmed cell death that occurs after the separation of normal cells from the extracellular matrix and neighboring cells. Evasion of anoikis improves the chances of survival of metastatic cancer cells, allowing the cancer cells to proliferate at new sites of attachment (Liotta and Kohn, 2004). Focal adhesion kinase (FAK) is a widely expressed protein tyrosine kinase that participates in the malignant invasion of tumors. Norepinephrine and E initiate Src-related FAK phosphorylation through ADRB2 and thus protect ovarian cancer cells from anoikis (Sood et al., 2010). Norepinephrine can also induce YAP1 dephosphorylation and nuclear translocation via ADRB2, thus protecting cervical cancer cells from anoikis (Li et al., 2020). Propranolol can also inhibit this NE-mediated process (Gong et al., 2019) (Figure 1). In addition to NE, neurotrophic factors and their ligands, such as BDNF/TrkB, can induce escape from anoikis in ovarian cancer, cervical cancer, and endometrial cancer cells (Yu et al., 2008; Bao et al., 2013; Yuan et al., 2018a).

## Dopamine

Dopamine is another catecholamine neurotransmitter and regulates various physiological functions of the central nervous system. Disorders related to the regulation of the dopamine system include Parkinson’s disease and schizophrenia. In a restraint stress model, intratumoural NE levels were found to remain elevated, whereas dopamine levels were dramatically decreased in the stress group compared with the control group (Moreno-Smith et al., 2011). The possible reason for the drop in the dopamine levels is that dopamine is a precursor for the synthesis of NE and E.

Norepinephrine-mediated tumor growth and angiogenesis were completely blocked with daily dopamine administration (Moreno-Smith et al., 2011) (Figure 1A). The signaling pathway that involves dopamine is the dopamine-mediated reversal of NE-induced Src phosphorylation. In addition, dopamine reduces the stress-mediated growth and microvessel density of ovarian cancer through tumor cell DR2 and inhibits the mobilization of endothelial progenitor cells from the bone marrow cavity into the peripheral circulation through DR2 on endothelial progenitor cells (Basu et al., 2001; Chakroborty et al., 2008; Moreno-Smith et al., 2011). In addition, dopamine can promote the maturation and normalization of the ovarian cancer vascular system through the DR1, allowing greater intake of chemotherapeutic drugs (Moreno-Smith et al., 2013). Based on these findings, dopamine replacement therapy may represent a novel treatment strategy to block the detrimental effects of chronic stress. Interestingly, the incidence of cancer in patients with schizophrenia may be lower than that in the general population (Mortensen, 1989; Barak et al., 2005; Asada et al., 2008; Chou et al., 2011). Patients with schizophrenia have high levels of the dopaminergic system, and preclinical studies have confirmed that dopamine can inhibit tumor angiogenesis. However, this view is still controversial, and it remains to be confirmed whether the lower incidence of cancer in schizophrenia patients is related to the hyperactivity of their dopaminergic system.

## Neurotrophic Factors

Neurotrophic factors are protein molecules that are necessary for the growth and survival of nerve cells. Neurotrophic factors belong to the small polypeptide growth factor family composed of five members: nerve growth factor (NGF), brain-derived neurotrophic factor (BDNF), neurotrophic factor-3 (NT-3), neurotrophic factor-4/5 (NT-4/5), and neurotrophic factor-6 (NT-6). Neurotrophic factors interact with two types of receptors: p75 and Trk receptors. The Trk receptors are necessary for neurite growth and cell survival. Different Trk receptors bind to specific neurotrophic factors with high affinity: NGF binds to TrkA, BDNF, and NT4/5 bind to TrkB, and NT-3 binds to TrkC (Chao and Hempstead, 1995; Retamales-Ortega et al., 2017).

**FIGURE 2:**
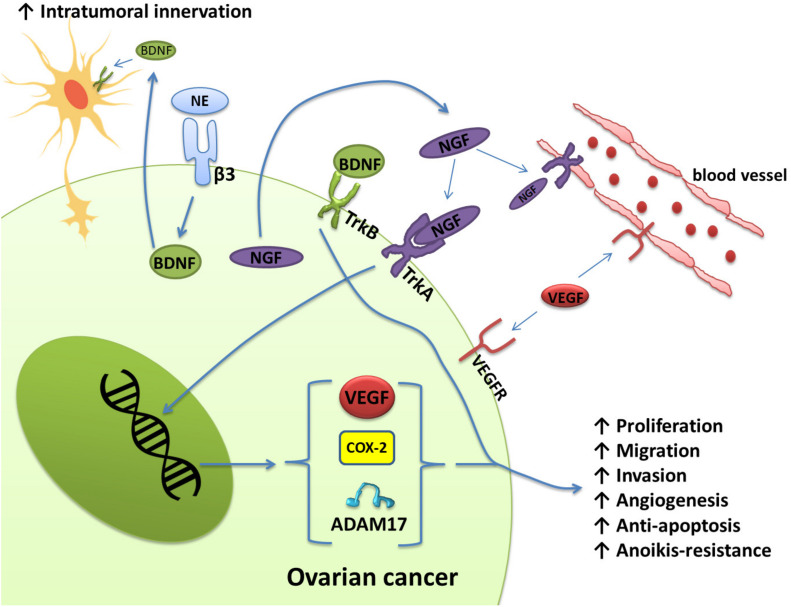
Schematic representation of the effects of NGF/TrkA and BDNF/TrkB, which are involved in several signaling pathways in ovarian cancer. Ovarian cancer cells express and secrete NGF. Through the activation of TrKA, NGF induces angiogenesis by directly stimulating the proliferation of endothelial cells. Nerve growth factor also regulates angiogenesis indirectly through the production of VEGF by ovarian cancer cells. In addition, NGF increases COX-2 levels, which induces the production of PGE-2. PGE-2 has been associated with invasion in cancer cells. ADAM17 also appears to be regulated by the activation of NGF/TrkA. Activation of the BDNF/TrkB pathway also confers migration, invasion, angiogenesis, and anoikis resistance to ovarian cancer cells. Norepinephrine can also bind to ADRB3 expressed by ovarian cancer cells to induce the production of BDNF, which then acts on TrkB receptors on nerve cells to increase the innervation of tumor tissues.

The expression levels of NGF and its receptor TrkA in ovarian cancer and cervical squamous cell carcinoma are significantly increased and related to the proliferation and metastasis of ovarian cancer as well as the clinical grade and nerve infiltration of cervical cancer (Tapia et al., 2011; Streiter et al., 2016; Retamales-Ortega et al., 2017; Long et al., 2018; Faulkner et al., 2020). Ovarian cancer cells express and secrete NGF, which directly stimulates endothelial cell proliferation by activating TrkA receptors to induce angiogenesis. Nerve growth factor also acts on the receptor TrkA on the surface of cancer cells in an autocrine manner to increase the protein expression levels of VEGF, COX-2, and A Disintegrin and Metalloproteinase 17 (ADAM17). These three proteins are related to angiogenesis, migration, and cell proliferation in epithelial ovarian cancer (Vera et al., 2014; Retamales-Ortega et al., 2017) (Figure 2). The activation of the receptor TrkB by BDNF also plays an important role in tumor progression. BDNF and TrkB are overexpressed in epithelial ovarian cancer tissues. Activation of the BDNF/TrkB pathway induces ovarian cancer cell migration, invasion, angiogenesis, and anoikis resistance (Qiu et al., 2006; Au et al., 2009; Siu et al., 2009). In addition to ovarian cancer, cervical cancer, endometrial cancer, and uterine leiomyosarcoma also exhibit high expression of BDNF and TrkB, which are closely related to adverse clinical phenomena, such as lymph node metastasis (Yu et al., 2008; Moon et al., 2011; Makino et al., 2012; Bao et al., 2013; Yuan et al., 2018a,b).

Moreover, neurotrophins released by tumor cells can stimulate adjacent nerve cells to develop nerve endings in the tumor. For example, NE can bind to ADRB3 expressed by ovarian cancer cells to produce BDNF, and then BDNF acts on TrkB receptors on host neurons to increase the innervation of the tumor (Entschladen et al., 2006; Allen et al., 2018) (Figure 2). These nerve endings may release catecholamines, which initiate the migratory and angiogenic activity of tumor cells, prerequisites for invasion and metastasis.

## Glucocorticoids

**FIGURE 3:**
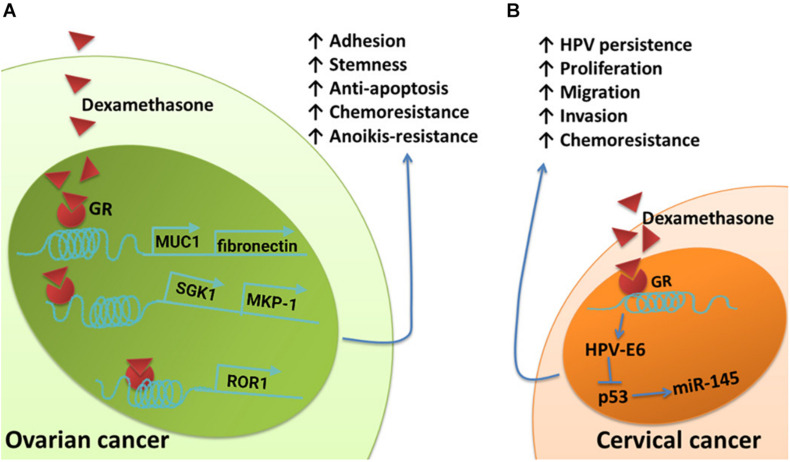
Schematic of the effects of glucocorticoids on pathways involved in cancer cell survival, metastasis, and chemoresistant signaling. **(A)** The upregulation of fibronectin and MUC1 induced by DEX contributes to DEX-induced pro-adhesion effects and protects ovarian cancer cells from chemotherapy. Dexamethasone induces increased expression of SGK1 and MKP-1, both of which promote cell survival. Dexamethasone induces anti-apoptotic features and drug resistance in ovarian cancer by promoting ROR1-mediated stemness. **(B)** Glucocorticoid-induced HPV–E6 expression effectively suppresses the upregulation of p53-dependent miR-145 and cellular apoptosis.

Glucocorticoids are another type of hormone that increase during a stress response. They are widely used clinically as anti-inflammatory and immunosuppressive agents. Glucocorticoids can also be used as adjuvant drugs with chemotherapy to reduce the side effects of chemotherapy. However, *in vitro* studies have demonstrated that glucocorticoids can promote tumor cell survival, metastasis, and drug resistance. The expression of receptor tyrosine kinase-like orphan receptor 1 (ROR1) is closely related to the phenotype of ovarian cancer stem cells, peritoneal metastasis, and the development of resistance to chemotherapy (Zhang et al., 2012; Zhang H. et al., 2014; Zhang S. et al., 2014; Henry et al., 2017; Karvonen et al., 2019). Dexamethasone (DEX), a synthetic glucocorticoid, can promote the expression of ROR1, fibronectin, and MUC1 by activating glucocorticoid receptors, thereby mediating stemness, adhesion, and drug resistance in cancer cells, respectively (Yin et al., 2016; Karvonen et al., 2020). The activation of glucocorticoid receptors can also upregulate the expression of serum and glucocorticoid-regulated kinase 1 (SGK1) and MKP-1, both of which can promote the survival of ovarian cancer cells (Melhem et al., 2009; Stringer-Reasor et al., 2015) (Figure 3A). Glucocorticoids can also affect the life cycle of HPV, interfere with the function of p53, and reduce the expression of miR-145, thus playing direct roles in the persistence of HPV infection and resistance to chemotherapy in cervical cancer patients (Feng et al., 2012; Shi et al., 2012) (Figure 3B).

As a common drug used for abortion in clinical practice, mifepristone has anti-glucocorticoid activity separate from its anti-progesterone effect. The addition of mifepristone to a combination cisplatin and paclitaxel regimen can prevent the development of drug resistance in ovarian cancer cells and cervical cancer cells (Jurado et al., 2009; Gamarra-Luques et al., 2012; Ponandai-Srinivasan et al., 2019). This also suggests that the activation of the glucocorticoid signaling pathway negatively impacts gynecological cancers.

Consistency between preclinical and clinical studies on ovarian cancer supports the hypothesis that glucocorticoid signaling has a promotive effect on solid tumors. However, cervical cancer patients with higher expression of glucocorticoid receptors have longer progression-free survival and overall survival (Block et al., 2017; Kost et al., 2019). The reason for the contradiction between clinical and experimental studies on cervical cancer is unclear, and whether other signaling pathways are involved remains to be studied.

## Tumor Immune Microenvironment

**FIGURE 4:**
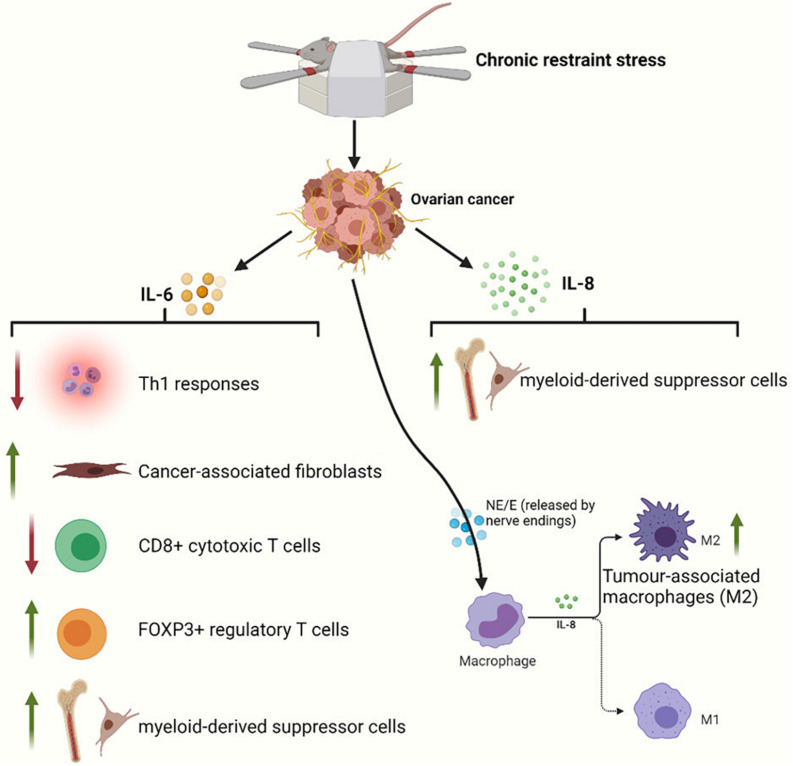
Restraint stress can act through a variety of immune mechanisms to promote tumor progression. IL-6 released by ovarian cancer cells can inhibit adaptive antitumor immunity by suppressing Th1 responses and CD8 + T cell activation and by driving and recruiting regulatory T cells. IL-6 also initiates cancer-associated fibroblast and MDSC infiltration of the tumor microenvironment. IL-8 released by ovarian cancer cells can inhibit innate immunity by polarizing macrophages toward a type 2 tumor-associated phenotype and by supporting MDSCs into the tumor microenvironment. NE and E also exacerbate the infiltration of M2 macrophages via β2-adrenergic receptors on macrophages.

It is clear that the tumor microenvironment, which is composed of a series of stromal cells [including macrophages, T cells, myeloid-derived suppressor cells (MDSCs), and fibroblasts] and their secreted products, has a significant impact on cancer progression. In this section, we will briefly discuss the effects of sustained stress on the immune microenvironment of gynecological cancers. A previous section explains that NE can induce the production of IL-6 and IL-8 in ovarian cancer cells and promote angiogenesis and metastasis. Additional effects of IL-6 include attenuation of Th1 responses in the tumor microenvironment (Johnson et al., 2018; Tsukamoto et al., 2018), activation of cancer-associated fibroblasts (Karakasheva et al., 2018), reductions in CD8 + cytotoxic T lymphocyte populations, increases in immunosuppressive FOXP3 + regulatory T cell populations (Kato et al., 2018), and enhanced generation of MDSCs (Hanazawa et al., 2018). In combination with chemotherapy, propranolol potentially results in improvements in circulating CD8 + T cells (Ramondetta et al., 2019). IL-8 also has a strong ability to recruit macrophages or MDSCs to the tumor microenvironment (Fousek et al., 2021). Macrophages have two different phenotypes: a tumor-suppressive phenotype (M1) and a tumor-supportive phenotype (M2). Tumor-associated macrophages (TAMs) mainly exhibit M2 characteristics. IL-8 can polarize macrophages toward the CD163 + M2 phenotype, which may contribute to poor survival in ovarian cancer (Ning et al., 2018). At the same time, stress hormones can also directly bind to β2-adrenergic receptors on the surface of macrophages (Sloan et al., 2010; Allen et al., 2018; Colon-Echevarria et al., 2020). Ultimately, this will exacerbate the infiltration of TAMs (Figure 4). In a study, treatment of mice with hexamethonium bromide resulted in a marked reduction in macrophage infiltration. In contrast, cytisine, a neuronal nicotinic acetylcholine (nACh) receptor agonist, could mimic the effects of restraint stress on macrophage infiltration (Allen et al., 2018). Therefore, macrophage infiltration mediates stress-enhanced progression.

## Clinical Trials

As mentioned above, several experiments have confirmed that the activation of β-adrenergic receptors can promote the malignant progression of ovarian cancer. However, the existing clinical research results are still conflicting. Some studies have reported that patients with epithelial ovarian cancer who used β-blockers have a lower chance of death and longer overall survival than patients who did not use β-blockers (Diaz et al., 2012; Al-Niaimi et al., 2016; Ramondetta et al., 2019). In contrast, other clinical studies have observed no association between the use of β-blockers and a reduction in ovarian cancer mortality (Heitz et al., 2013; Johannesdottir et al., 2013; Cho et al., 2020). One study even reported that patients who used β-blockers during the perioperative period had an increased risk of death (Gonzalez et al., 2020). Notably, almost all patients in the above studies were using selective β1-receptor blockers, but it is more likely that non-selective β-blockers can benefit patients with ovarian cancer. However, the use of non-selective β-blockers has been limited due to well-known side effects. Hence, these contradictory research results highlight the importance of stratification studies based on the type of β-blocker. Otherwise, the results are unreliable (Hefner and Csef, 2016). After categorizing the selectivity of β-blockers, we observed that ovarian cancer patients who used non-selective β-blockers showed reduced cancer-specific mortality. Selective β-blocker intake did not affect prognosis and even produced reduced overall survival (Watkins et al., 2015; Heitz et al., 2017; Harding et al., 2019). The reason underlying this finding is still unclear. However, the patients taking selective β-blockers tended to be older and have various chronic underlying diseases, which might make them more intolerant of cancer therapy.

Glucocorticoids have been included in standard treatment plans because they can reduce the side effects of chemotherapy. However, an increasing number of experiments have proven that glucocorticoids can promote the survival of tumor cells. These results have caused concerns among clinicians to some extent, resulting in the question: Is the adjuvant application of glucocorticoids safe during chemotherapy? However, when DEX is used during the perioperative period or chemotherapy administration, there is currently no evidence to indicate that the application of this glucocorticoid will negatively impact the prognosis of patients (Munstedt et al., 2004; De Oliveira et al., 2014; Djedovic et al., 2018). We have yet to determine whether the small sample size affected the results of the study or whether the benefits of glucocorticoids, such as an increased white blood cell count and increased patient compliance, concealed its protective effect on tumor cells. In general, before further research is performed to address this question, we should at least allay fears related to the use of glucocorticoids; after all, their benefits are obvious.

## Discussion

Several preclinical experiments have demonstrated overexpression of stress hormone receptors in ovarian cancer cells and cervical cancer cells. Various stress hormones produced under chronic stress exert protective effects on cancer cells through these receptors, which eventually leads to adverse clinical results. Simultaneously, cancer cells can also initiate their own innervation by releasing neurotrophic factors. Under chronic stress, these nerve endings release stress hormones (mainly NE and E), which in turn bind to the overexpressed receptors on tumor cells and induce various effects (Faulkner et al., 2019). Therefore, it is theoretically feasible to try to eliminate tumor innervation or block stress hormone receptors on the surface of tumor cells. Drugs that block these receptors are common in clinical treatment and therefore have the greatest potential. However, the relatively small cohort of studies evaluating non-selective β-blockers have led us to question the effectiveness of these drugs in treating cancers. Hence, whether to use non-selective β-blockers in gynecological cancer patients has not yet been determined. Likewise, there is no sufficient evidence indicating that using glucocorticoids will shorten the lifespan of chemotherapy-treated patients. Therefore, we do not support the aversion to using DEX for gynecological cancer treatment; after all, several preliminary studies have demonstrated that DEX is effective in preventing postoperative nausea, vomiting, and the side effects of chemotherapy. Dopamine and DR agonists are widely used in the treatment of Parkinson’s disease, hyperprolactinemia, and other non-neoplastic diseases; they are inexpensive and have few side effects. Therefore, the prospect of dopamine being used to treat cancer patients in the future is also very encouraging.

In summary, we should view a tumor as a complete organism. This “organism” contains tumor cells, stromal cells, and vascular and neural connections to its host. This provides not only mechanisms for disease progression but also opportunities for therapeutic intervention. Further studies are needed to clarify the exact relationships between PNI and stress hormones in gynecological cancers. Only through this work can the process of using these inexpensive drugs to treat gynecological cancers be accelerated.

## Author Contributions

GC and LQ contributed equally to this manuscript. GC contributed to conception and design of the study and wrote the first draft of the manuscript. LQ screened the relevant literature and revised the manuscript. All authors contributed to the article and approved the submitted version.

## Conflict of Interest

The authors declare that the research was conducted in the absence of any commercial or financial relationships that could be construed as a potential conflict of interest.

## Abbreviations

SNS: sympathetic nervous system
PNI: perineural invasion
NE: noradrenaline
E: epinephrine
DR: dopamine receptor
PGE2: prostaglandin E2
MMPs: metalloproteinases
MMP-2: metalloproteinase 2
MMP-9: metalloproteinase 9
IL-6: interleukin 6
IL-8: interleukin 8
VEGF: vascular endothelial growth factor
ADAM17: A Disintegrin and Metalloproteinase 17
DEX: dexamethasone
MDSCs: myeloid-derived suppressor cells
TAMs: tumor -associated macrophages.
